# Effect of defocus incorporated multiple segments (DIMS) spectacle lenses on myopia progression in children: a retrospective analysis in a German real-life clinical setting

**DOI:** 10.1186/s12886-024-03666-5

**Published:** 2024-09-12

**Authors:** Birte Neller, Kai Neller, Hartmut Schwahn, Ann-Isabel Mattern, Machteld Devenijn, Achim Langenbucher, Berthold Seitz, Hakan Kaymak

**Affiliations:** 1 Internationale Innovative Ophthalmochirurgie, Breyer Kaymak Klabe Augenchirurgie, Düsseldorf, Germany; 2 Institute of Experimental Ophthalmology, Saarland University, Homburg, Germany; 3 Department of Ophthalmology, Saarland University Medical Center UKS, Homburg, Germany; 4 Gottfried O.H. Naumann-Institute of Epidemiology and Prevention of Myopia, Saarland University, Homburg, Germany; 5 MVZ Makula-Netzhaut-Zentrum Breyer Kaymak Klabe, Theo-Champion-Str. 1, D-40549 Duesseldorf, Germany

**Keywords:** Myopia, Axial length, Children, Defocus incorporated multiple segments (DIMS), Spectacle lenses

## Abstract

**Objectives:**

This retrospective analysis evaluates the treatment success of “Defocus Incorporated Multiple Segments” (DIMS) spectacle lenses in a real-life clinical setting in Germany.

**Materials and methods:**

Axial length (AL) and objective refraction of 166 eyes treated with DIMS at baseline and 12-month follow-up were analyzed. Annual AL growth rate within the range of physiological growth rate was considered a successful treatment. Myopia progression of ≥ -0.5 D/yr accounted as treatment success. Differences in percentages of treatment success of subgroups depending on baseline AL and age against treatment success of the total population were investigated.

**Results:**

Considering all eyes, treatment success regarding AL growth and myopia progression was achieved in 46% and 65%, respectively. Male eyes with moderate AL showed treatment success in a higher proportion (73%, *p* < 0.01; 89%, *p* < 0.01); eyes with high AL showed treatment success in a lower proportion (25%, *p* < 0.01; 51%, n.s.). Female eyes showed the same trend but without statistical significance (moderate AL: 49%; 68%; high AL: 40%; 62%). Younger children showed treatment success in a lower proportion (male: 11%, *p* < 0.01; 38%, *p* < 0.05; female: 25%, *p* < 0.01; 42%, *p* < 0.01). Older children showed treatment success in a higher proportion (male: 60%, *p* < 0.05; 78% *p* < 0.05; female: 53%, n.s.; 77% *p* < 0.05).

**Conclusions:**

Eyes with moderate baseline AL and of older children showed treatment success after 12 months of DIMS treatment. Eyes with a high baseline AL and of younger children showed treatment success in a smaller proportion, therefore combination treatment should be considered. In future studies, males and females should be assessed separately.

**Supplementary Information:**

The online version contains supplementary material available at 10.1186/s12886-024-03666-5.

## Introduction

Myopia usually starts in childhood and, in most cases, progresses until young adulthood [1]. Myopia is caused by an imbalance between refractive power and axial length of the eye and, in most cases, is attributable to an excessive axial length growth (axial myopia). As the axial length of the eye increases, so does the risk of serious complications such as glaucoma, retinal detachment, and choroidal neovascularization [2–4]. According to estimates, by 2050 10% of the world’s population could be highly myopic (more myopic than − 5 D), entailing a multiplicative risk of these serious diseases [5]. An increasing prevalence of myopia was also observed in Europe, particularly among young adults [6, 7]. Strategies and various treatment options have been developed to inhibit myopia progression (“myopia treatment”), including pharmacological treatment with atropine eye drops, orthokeratology contact lenses, rigid and soft contact lenses as well as bifocal, multi-segmented, and progressive spectacle lenses [8]. Promising non-invasive treatment options include spectacle lenses with “Defocus Incorporated Multiple Segments” (DIMS) where a single vision lens base carries a multiple positive power additions arranged in a “multi-segmented” fashion. More details on the mode of therapeutic action, the design and optical characteristics of DIMS spectacle lenses can be found in the literature [9–12]. Practically speaking, DIMS spectacle lenses correct myopia and, at the same time, treat myopia in a fully non-invasive manner, which makes them an attractive option for myopia treatment. DIMS spectacle lenses have been proven in RCTs to be highly efficient in inhibiting myopia progression, with 60% less axia length growth in the myopic treatment group over a myopic untreated group [10, 13, 14]. Compared to the average axial length growth rate of eyes of a cohort of children not becoming myopic but remaining emmetropic, treatment with DIMS spectacle lenses, on average, restored physiological axial length growth rate, which we considered a treatment success [15].

General safety and ease of acceptance of DIMS spectacle lenses had previously been evaluated in clinical studies with Chinese children, and in knowing that these lenses can inhibit the progression of myopia, 90% of the children would have chosen the DIMS spectacle lenses (again) [16]. After 2 years of continuous wear, Lam et al. did not find any significant difference in various visual functions of children who wore DIMS spectacle lenses and those who wore single vision lenses [17]. In an experimental setting to assess the worst possible scenario, young myopic adults were forced to gaze only through the characteristic peripheral defocusing zone of the DIMS spectacle lenses. Although some reduction in visual acuity and in contrast sensitivity could be recorded, these effects were of no clinical, and thus practical, relevance [18, 19]. Noteworthy, in the cohort of young myopic adults, additional glare did not significantly reduce contrast sensitivity or visual acuity with DIMS spectacle lenses, even when combined with low-dose atropine [18, 19].

Since April 2021, DIMS spectacle lenses are available on the market in Germany, Austria, and Switzerland under the brand name “MiYOSMART^®^” (Hoya Lens Thailand Ltd., Bangkok, Thailand). This retrospective data analysis aims to evaluate the effect of DIMS spectacle lenses on myopia progression in a cohort of progressively myopic children after the first year of treatment outside a RCT, but in a real-life clinical setting in our private practice in Düsseldorf, Germany.

As a measure of myopia progression, axial length growth is considered more useful than change in refraction [20]. However, since axial length growth is naturally strongly age-depending, a uniform limit for all ages is not applicable [21]. We have therefore established a routine to assess the therapeutic efficacy of the myopia treatment by means of comparing the actual annual axial length growth rate of a patient with the age-matched physiological growth rate. From the actual growth rate’s degree of approximation to the physiological growth rate, conclusions on the efficacy of the current treatment can be drawn [15, 22]. Based on this approach, we propose a simple color-coded scheme to classify the success of myopia treatments and here to assess the efficacy of the first year of treatment with DIMS spectacles. This evaluation allows the assessment of treatment efficacy without the need for an untreated control group, which increasingly poses an ethical dilemma [23, 24].

Epidemiological studies have found that axial lengths in the highest percentile (98th percentile) are associated with a 100% risk of myopia and a 31% (male eyes) and 43% (female eyes) risk of high myopia [25]. Further there is uncertainty whether respondence to myopia treatments depends on the extent of axial length and/or refractive myopia of children’s eye [26]. For these two reasons, we considered the therapeutic efficacy of DIMS spectacle lenses separately for eyes with moderately increased axial lengths and eyes with high axial lengths.

Through our own experience, we had gained the impression that younger children respond less well to myopia treatments. Therefore, we considered treatment efficacy also separately for children older and younger than 10 years at baseline. In Germany, the age of 10 marks approximately the time when children move from primary to secondary school and thus the time when the effects of school entry combined with frequent close work should have already occurred.

## Materials and methods

### Patient cohort and intervention

In our practice, myopia treatment is generally recommended to myopic children, if the axial length of at least one eye is above the 50th percentile of the sex-specific reference curve by Truckenbrod et al. [27], because the axial length of an eye at the 50th percentile is associated with emmetropia in adulthood (male: 23.77 mm, female: 23.33 mm). We allow all patients, recommended for treatment, to choose, according to their preference, from a variety of myopia treatment options: low-dose atropine treatment [28], DIMS spectacles [10, 13, 14], multifocal contact lenses [29, 30] and orthokeratology lenses [31]. To patients opting for DIMS spectacles, the new prescription for DIMS spectacle lenses is given at that initial (“baseline”) visit, if needed. It is in the parents’ responsibility to have the prescribed DIMS spectacles made for their child as soon as possible. Patients are advised to wear the DIMS spectacle lenses for all waking hours and not to use any other optical correction (except short-term wearing of soft contact lenses for sports if applicable).

For the present study, a search of the patient records of all myopic children who visited our practice between April 2021 and January 2023 was conducted. Children who met the following criteria were included:

- follow-up visit after 10 to 15 months after baseline (“12-month follow-up”).
- a minimum 10 months of continuous wear of DIMS spectacle lenses prior to 12-month follow-up.
- valid axial length measurements recorded for both, baseline visit and 12-month follow-up.

### Procedures and examinations

For already myopic children, we routinely use axial length, measured with IOLMaster 700 (Carl Zeiss Meditec, Germany), as the main indicator of myopia and myopia progression. Objective refraction is performed with an autorefractometer (KR-800 S, Topcon, Japan) without cycloplegia. Subjective refraction is performed if visual acuity of 0.0 logMAR is not achieved with current correction or the child expresses problems with the current correction. Consequently, new prescription results from the subjective refraction (usually in case of myopia progression of -0.5 D and more). As part of our internal quality management, we have analyzed the discrepancy between cycloplegic and non-cycloplegic autorefraction and found a mean difference of -0.12 ± 0.21 D, *n* = 42 (data unpublished). This difference was considered clinically not significant. Accordingly, cycloplegia is not used as standard and is only applied in children where axial length growth and change in refraction do not match, or in children suspected of showing accommodation during refraction.

### Assessment of axial length growth rate and myopia progression

To monitor the efficacy of the myopia therapies, we have established a routine by means of comparing the patient’s annual axial length growth (“AL growth”) rate with the average physiological AL growth rate of an age-matched cohort of children who become and remain emmetropic (hereafter “physiological axial length growth rate”). The annual AL growth rate [mm/yr] was calculated from the patient’s two valid axial length measurements (at baseline and at the 12-month follow-up visit), normalized to an exact 12-month interval by adjusting to the actual time elapsed between the two measurements. From the AL growth rate’s degree of agreement to the physiological axial growth rate a conclusion on the efficacy of the current treatment can be drawn [15, 22]. Specifically, we chose to plot the AL growth rate against the age in a simplified nomogram and to categorize the AL growth rates into three distinct categories: the “green zone” reflects an uncritical AL growth rate that corresponds (within a 25% agreement limit) to the physiological AL growth rate, which we consider to be the goal of any myopia treatment. The “yellow zone” reflects a moderately excessive AL growth rate, which is greater than + 25% but less than + 50% above the age-matched physiological AL growth rate. The “red zone” reflects a highly excessive AL growth rate, corresponding to more than + 50% above the age-matched physiological AL growth. To calculate the physiological AL growth rate epidemiologic data by Truckenbrod et al. [27] and own data collection of emmetropic schoolchildren in Germany [32] was used. Male and female eyes were evaluated separately, as they have different normative data concerning ocular development and therefore physiological AL growth rate. We note that for mere practical reasons and to avoid a misleading seeming accuracy, the lower limit of AL growth rate is set at 0.10 mm/yr as this reflects the level of agreement for repeated axial length measurements with currently available biometers [33].

According to this color-coded scheme, a treatment effect (hereafter “treatment success”) was acknowledged for assessed AL growth rates that fell within the “green zone” of the nomogram; whereas for AL growth rates that were located in the “red zone” or the “yellow zone” no or only insufficient treatment effect could be acknowledged.

Myopia progression (change in spherical equivalent) was also normalized to a 12-month interval [D/yr] by adjusting for the respective individual time intervals between the objective refraction measurements. Myopia progression was divided into “no and low progression” (up to and including − 0.5 D/yr) and “moderate and high progression” (more than − 0.5 D/yr) [34, 35]. No or low myopia progression was considered a treatment success, while moderate or high progression was classified as no or only insufficient treatment effect.

### Statistics

To assess potential differences in treatment efficacy, the percentage treatment success of eight subgroups:

1. male eyes with a moderate baseline AL below the 98th percentile of the reference curve,
2. female eyes with a moderate baseline AL below the 98th percentile,
3. male eyes with a high baseline AL above the 98th percentile,
4. female eyes with a high baseline AL above the 98th percentile,
5. male eyes younger than 10 years at baseline,
6. female eyes younger than 10 years at baseline,
7. male eyes older than 10 years at baseline, and.
8. female eyes older than 10 years at baseline.

were tested against the overall percentage of treatment success of the total population using one-sided binomial tests, using statistics program “R” (version 4.0.3). The criterion for statistical significance was *p* < 0.05.

### Ethical approval and patient informed consent

The need for ethical approval was waived by the ethics committee of Ärztekammer Nordrhein for this kind of retrospective, descriptive, non-interventional study. All procedures performed were in accordance with the Helsinki Declaration of 1964 and its later amendments or comparable ethical standards. The data analyzed were routinely collected during treatment, and no additional interventions or examinations were performed. Informed consent was obtained from the legal guardians of all individual participants included in the study to analyze the routinely collected data anonymously for scientific purposes.

## Results

### Patient characteristics

Database search revealed that most young myopic patients who started myopia treatment in our practice chose the DIMS spectacle lenses as their treatment option over the alternatives offered to them. From April 2021 to January 2023, 321 children were recommended to start wearing DIMS spectacle lenses. Of these, 83 children with 166 eyes (66 eyes of 33 boys and 100 eyes of 50 girls) met the above-mentioned criteria so that the annual growth rate under treatment with DIMS spectacle lenses could be calculated.

Table 1 shows baseline characteristics for all eyes, separately for male and female eyes. Baseline characteristics of the analyzed eyes of the eight subgroups divided based on baseline axial length and age are shown in supplementary table 1. Of note, 13 of 83 (16%) children were of Asian ethnicity (male: 6 of 33, 18%; female: 7 of 50, 14%); all others were caucasian.

**Table 1:** Baseline characteristics of analyzed eyes (separately for male and female eyes)

**male (** ***n*** ** = 66 eyes of 33 boys)**	age [years]	axial length [mm]	spherical equivalent [D]
range	6.4 to 15.2	23.50 to 28.01	-7.00 to -1.25
mean ± SD	11.4 ± 2.6	25.27 ± 1.00	-3.94 ± 1.49
median	11.5	25.16	-4.06
**female (** ***n*** ** = 100 eyes of 50 girls)**	age [years]	axial length [mm]	spherical equivalent [D]
range	7.2 to 16.9	23.28 to 26.32	-8.75 to -0.625
mean ± SD	10.9 ± 2.5	24.63 ± 0.72	-3.88 ± 1.57
median	10.7	24.56	-3.75

**Fig. 1:**
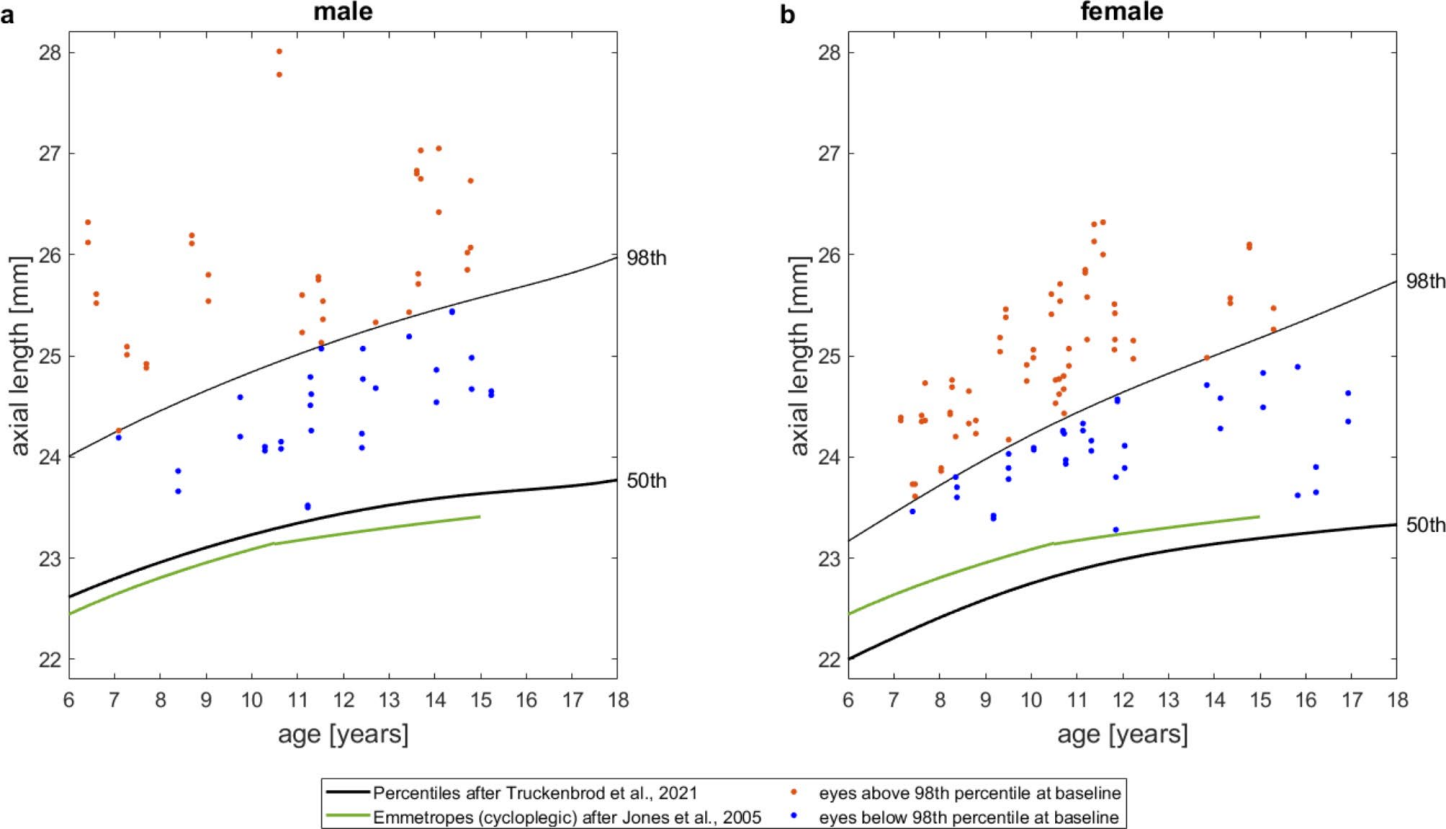
Baseline axial lengths plotted against age for each eye (blue: baseline AL below the 98th percentile, orange: baseline AL above the 98th percentile), overlaid by the 98th and 50th percentiles of axial lengths as assessed by Truckenbrod et al. 27 (black) and the modeled AL growth for emmetropic children of the OLSM as assessed by Jones et al. 36 (green); **a**: male eyes. **b**: female eyes

Figure 1 shows the baseline axial lengths (AL) plotted against the individual age with reference to the 98th and 50th percentiles curves of a German cohort as collected by Truckenbrod et al. [27] (black lines) and to the modeled AL growth for emmetropic children of the OLSM (Orinda Longitudinal Study of Myopia) as assessed by Jones et al. [36] from cycloplegic autorefraction. All baseline values lie above the 50th percentile, which corresponds to axial length associated with emmetropia in adulthood, and also above the modeled AL growth curve for emmetropic children of Jones et al. [36]. More than half of baseline AL are above the 98th percentile, which represents axial length associated with high myopia in adulthood, in both the male (55%) and female (63%) cohort.

**Fig. 2:**
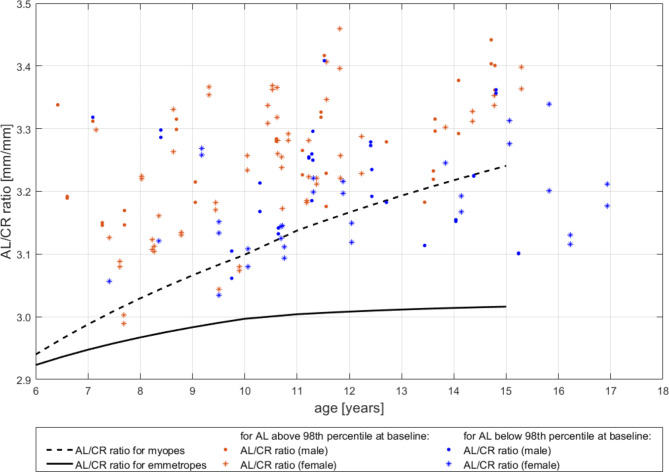
Baseline AL/CR ratio plotted against age for each eye (blue baseline AL below 98th percentile, orange: baseline AL above 98th percentile; dots: male eyes, asterisks: female eyes), overlaid by development of AL/CR ratio as calculated from the data by Jones et al. 36 for emmetropic eyes (solid black line) and for myopic eyes (dotted black line)

Figure 2 depicts the AL/CR ratios of all eyes at baseline as plotted against the individual age, overlaid with AL/CR ratio development as calculated from model curves for children who remained emmetropic (solid black line) and myopic children (dotted black line) by Jones et al. [36]. It reveals that the baseline AL/CR ratios of the analyzed eyes are higher than those of the emmetropes, indicating a relatively increased AL of these eyes and confirming the categorization of axial myopia.

The mean follow-up visit was 12.6 ± 1.1 (mean ± SD) months after the baseline visit. At the time of the follow-up visit the children had worn their spectacle lenses, on average, for 11.7 ± 0.9 months. A review of patient record revealed that after a few days of adaptation, the DIMS spectacle lenses were generally well tolerated, and no complaints were recorded.

### Effect of DIMS spectacle lenses on axial length growth

**Fig. 3:**
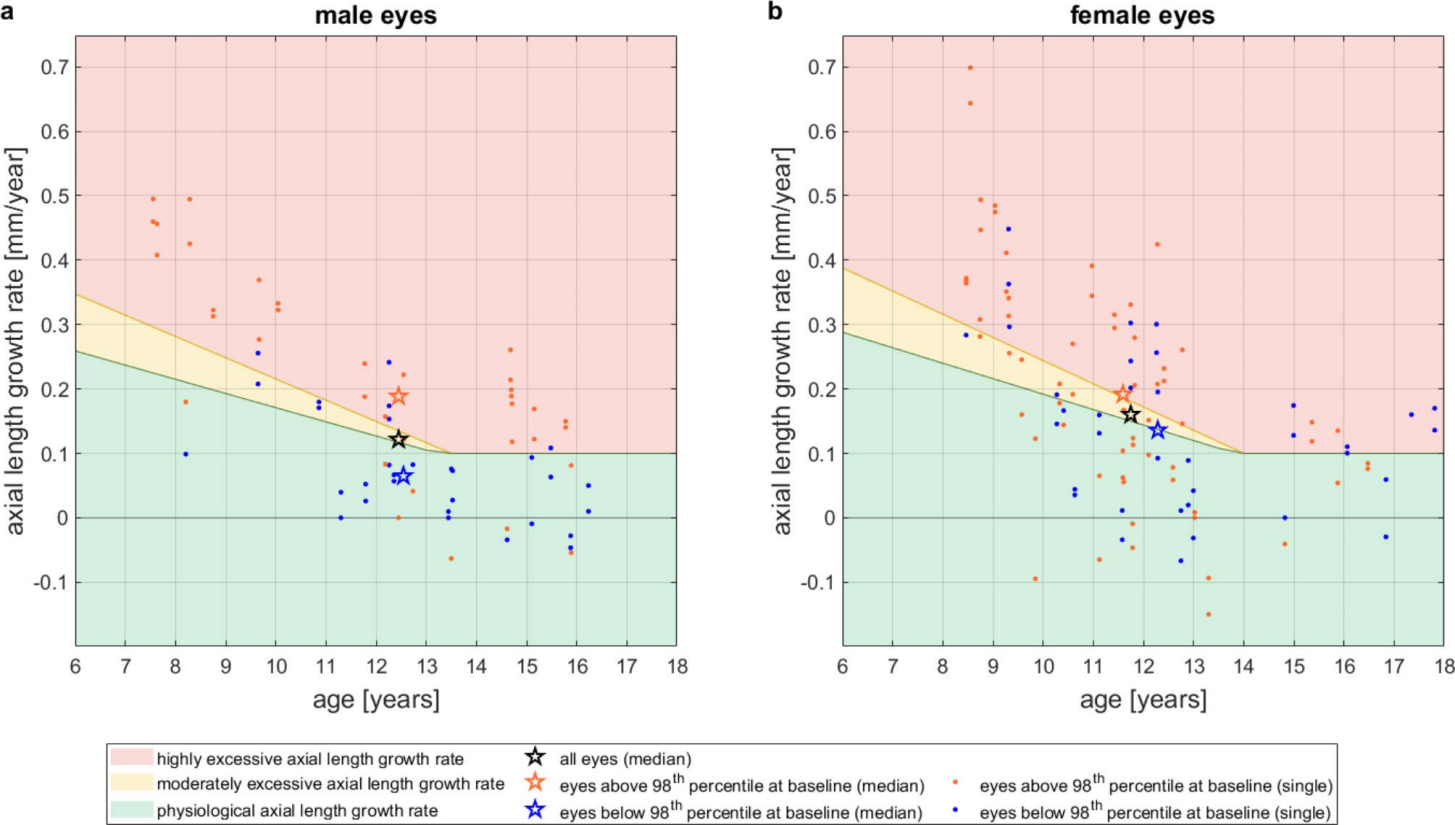
Individual, annual AL growth rates at 12-month follow-up visit (points) and respective median of age and AL growth rate (stars) according to baseline axial length; blue: eyes with baseline axial length below the 98th percentile, orange: eyes with baseline axial length above the 98th percentile, black: median of all eyes. **a**: male eyes. **b**: female eyes. In the overlaid color-coded zones, the “green zone” corresponds to the physiological AL growth rate (within a 25% agreement limit); the “yellow zone” reflects a moderately excessive AL growth rate, corresponding to more than + 25% but less than + 50% of the average physiological AL growth rate; the “red zone” reflects a highly excessive AL growth rate, corresponding to more than 50% above the physiological AL growth rate

In Fig. 3, the annual AL growth rates at 12-month follow-up visit of all eyes were plotted individually against the actual age as well as the respective median of age and AL growth rates separately for males (Fig. 3a) and females (Fig. 3b). The individual growth rates are color-coded based on the baseline axial length of the eye (below or above the 98th percentile); likewise the corresponding medians.

**Fig. 4:**
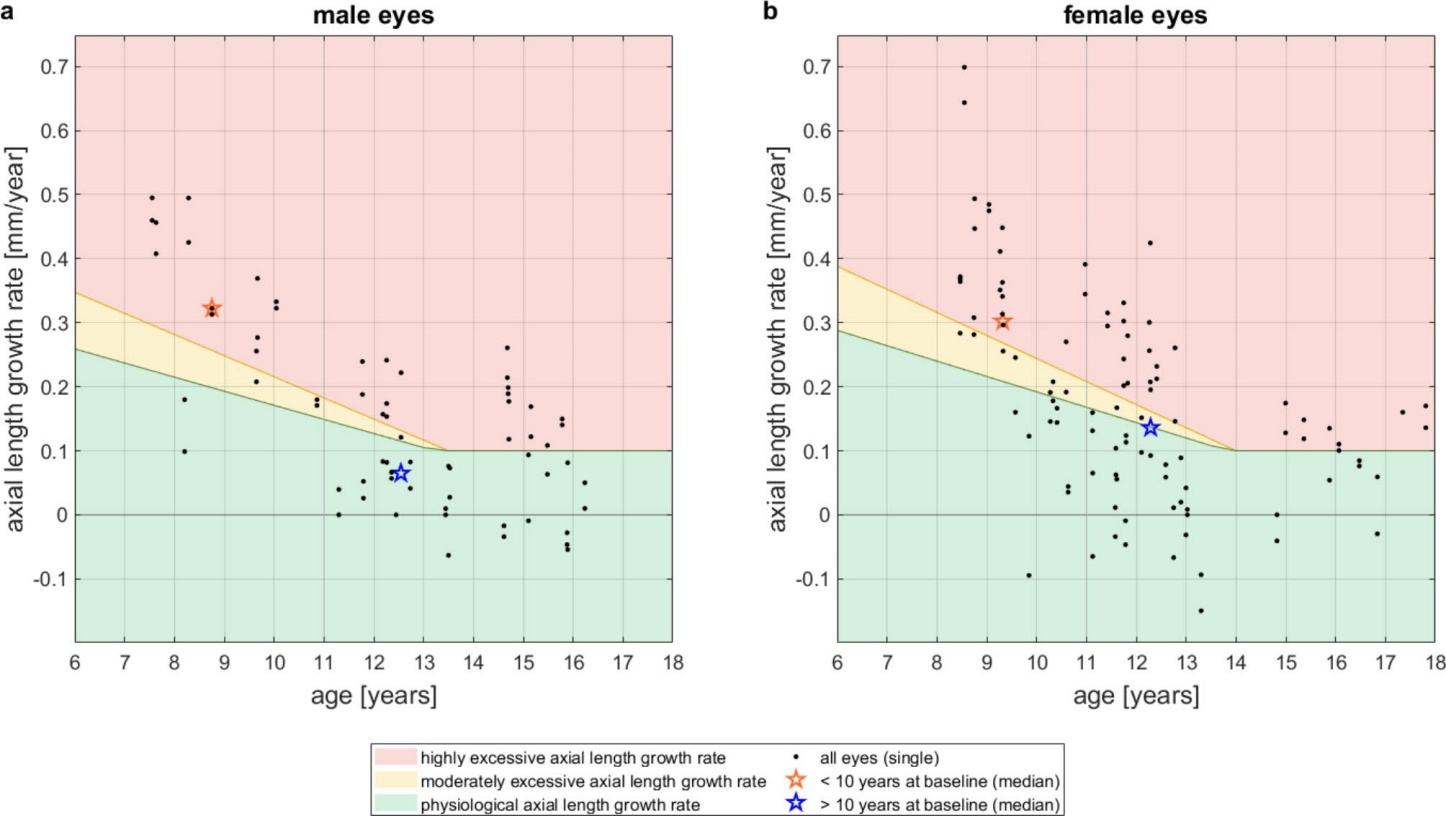
Individual, annual AL growth rates at 12-month follow-up visit (points) and respective median of age and AL growth rate (stars) for different age groups; orange: median of eyes younger than 10 years at baseline and thus younger than 11 years after one year of treatment, blue: median of eyes older than 10 years at baseline and thus older than 11 years after one year of treatment, black: all eyes. **a**: male eyes. **b**: female eyes. In the overlaid color-coded zones, the “green zone” corresponds to the physiological AL growth rate (within a 25% agreement limit); the “yellow zone” reflects a moderately excessive AL growth rate, corresponding to more than + 25% but less than + 50% of the average physiological AL growth rate; the “red zone” reflects a highly excessive AL growth rate, corresponding to more than 50% above the physiological AL growth rate

Figure 4shows medians of age and AL growth rates for the two different age groups (younger/older than 10 years at baseline and thus younger/older than 11 years after one year of treatment) separately for males (Fig. 4a) and females (Fig. 4b).

Table 2Lists the percentages of AL growth rates as classified according to the color-coded nomogram (Figs. 3 and 4). Within the total population of 166 eyes, 46% had notable treatment success, i.e., physiological AL growth rate (“green zone”).

**Table 2:** Percentages of AL growth rates after one year of treatment with DIMS spectacle lenses according to the color-coded nomogram for all eyes and for subgroups of eyes with baseline axial length below or above the 98th percentile of reference curves [17] (Fig. 3) and for subgroups of eyes younger or older than 10 years at baseline and thus younger or older than 11 years after one year of treatment (Fig. 4)

**all eyes**			
	**male**	**female**	**total**
highly excessive AL growth rate (red zone)	47%	48%	46%
moderately excessive AL growth rate (yellow zone)	6%	9%	8%
physiological AL growth rate (green zone)	47%	43%	46%
**moderate baseline axial lengths (below the 98th percentile)**			
	**male (** ***n*** ** = 30)**	**female (** *n* ** = 37)**	
highly excessive AL growth rate (red zone)	17%	46%	
moderately excessive AL growth rate (yellow zone)	10%	5%	
physiological AL growth rate (green zone)	73%	49%	
	*p* < 0.01	*n.s.*	
**high baseline axial lengths (above the 98th percentile)**			
	**male (** ***n*** ** = 36)**	**female (** ***n*** ** = 63)**	
highly excessive AL growth rate (red zone)	72%	49%	
moderately excessive AL growth rate (yellow zone)	3%	11%	
physiological AL growth rate (green zone)	25%	40%	
	*p* < 0.01	*n.s.*	
**younger than 10 years at baseline**			
	**male (** ***n*** ** = 18)**	**female (** ***n*** ** = 36)**	
highly excessive AL growth rate (red zone)	*72%*	*56%*	
moderately excessive AL growth rate (yellow zone)	*17%*	*19%*	
physiological AL growth rate (green zone)	*11%*	*25%*	
	*p* < 0.01	*p* < 0.01	
**older than 10 years at baseline**			
	**male (** ***n*** ** = 48)**	**female (** ***n*** ** = 64)**	
highly excessive AL growth rate (red zone)	*38%*	*44%*	
moderately excessive AL growth rate (yellow zone)	*2%*	*3%*	
physiological AL growth rate (green zone)	*60%*	*53%*	
	*p* < 0.05	*n.s.*	

Physiological AL growth rates (green zone) were accounted for successful treatment, moderately excessive AL growth rates (yellow zone) and highly excessive AL growth rates (red zone) accounted for insufficient treatment effect. The *p*-values correspond to the statistical binomial tests of the percentage of treatment success (green zone) in the eight cohorts against the percentage of treatment success (green zone) in the total population

One-sided binomial statistical analysis reveals that male eyes with moderate baseline AL had a significantly higher percentage of treatment success than the total population (73% > 46%, *p* < 0.01), while male eyes with high baseline AL had a significantly lower percentage of treatment success than the total population (25% < 46%, *p* < 0.01). Also, girls with moderate baseline AL showed a slightly higher percentage of treatment success than the total population (49% > 46%, n.s.), while girls with high baseline AL had a somewhat lower percentage of treatment success than the total population (40% < 46%, n.s.), but these differences were not statistically significant.

With regard to the two different age groups, binomial statistical analysis revealed significantly lower percentage of treatment success in both sexes for eyes of younger children compared to the population in both sexes (male: 11% < 46%, *p* < 0.01; female: 25% < 46%, *p* < 0.01). Eyes of older children showed slightly higher percentage of treatment success compared to total population (male: 60% > 46%, *p* < 0.05; female: 53% > 46%, n.s.).

Table 3lists the medians for age and AL growth rate for the respective eight cohorts as depicted in Figs. 3 and 4 and for all eyes. In the cohort of children with eyes with moderate baseline axial lengths, the median age of boys and girls differs by only 0.25 years (3 months), but the median AL growth rate of girls is more than twice the median rate of the boys (0.14 mm/yr vs. 0.06 mm/yr). Nevertheless, the median of AL growth rate of both sexes is in the “green zone”. In the cohort of children with eyes with high baseline AL, girls have the same median AL growth rate as boys (0.19 mm/yr) but are 0.86 years (10.3 months) younger. In the cohort of younger children, the median age of boys and girls differ by 0.57 years (6.8 months), with the girls being older. As therefore expected, female eyes showed lower median AL growth rate (0.30 mm/yr) than males (0.32 mm/yr). In the cohort of older children, the age median of boys and girls differ by 1.01 years, with the boys being older. As therefore expected, male eyes showed lower median AL growth rate (0.08 mm/yr) than females (0.11 mm/yr). For both male and female eyes, the median AL growth rate is in the “green zone”.

**Table 3:** Median of age and annual AL growth rates corresponding to Figs. 2 and 3

**all eyes**		
	**male**	**female**
age (median) [yrs]	12.45	11.75
axial length growth rate (median) [mm/yr]	0.12	0.16
**moderate baseline axial lengths (below the 98th percentile)**		
	**male (** ***n*** ** = 30)**	**female (** ***n*** ** = 37)**
age (median) [yrs]	12.54	12.29
axial length growth rate (median) [mm/yr]	0.06	0.14
**high baseline axial lengths (above the 98th percentile)**		
	**male (** ***n*** ** = 36)**	**female (** ***n*** ** = 63)**
age (median) [yrs]	12.45	11.59
axial length growth rate (median) [mm/yr]	0.19	0.19
**younger than 10 years at baseline**		
	**male (** ***n*** ** = 18)**	**female (** ***n*** ** = 36)**
age (median) [yrs]	8.75	9.32
axial length growth rate (median) [mm/yr]	0.32	0.30
**older than 10 years at baseline**		
	**male (** ***n*** ** = 48)**	**female (** ***n*** ** = 64)**
age (median) [yrs]	13.51	12.50
axial length growth rate (median) [mm/yr]	0.08	0.11

### Effect of DIMS spectacle lenses on myopia progression

Table 4lists the proportions of myopia progression (change in spherical equivalent) up to and including − 0.5 D/yr (“no and low progression”) and greater than − 0.5 D/yr (“moderate and high progression”) after 12 months of treatment.

**Table 4:** Percentages of myopia progression (change in spherical equivalent) after one year of treatment with DIMS spectacle lenses, categorized into moderate and high progression (> 0.5 D/yr) and non and low progression (≤ 0.5 D/yr) for subgroups of eyes with baseline axial length below or above the 98th percentile of reference curves [17] and for subgroups of eyes younger or older than 10 years at baseline (thus younger or older than 11 years after one year of treatment)

**all eyes**			
	**male**	**female**	**total**
moderate and high progression (> 0.5 D/yr)	32%	36%	35%
no and low progression (≤ 0.5 D/yr)	68%	64%	65%
**moderate baseline axial lengths (below the 98th percentile)**			
	**male (** ***n*** ** = 27)**	**female (** ***n*** ** = 37)**	
moderate and high progression (> 0.5 D/yr)	11%	32%	
no and low progression (≤ 0.5 D/yr)	89%	68%	
	*p* < 0.01	*n.s.*	
**high baseline axial lengths (above the 98th percentile)**			
	**male (** ***n*** ** = 35)**	**female (** ***n*** ** = 63)**	
moderate and high progression (> 0.5 D/yr)	49%	38%	
no and low progression (≤ 0.5 D/yr)	51%	62%	
	*n.s.*	*n.s.*	
**younger than 10 years at baseline**			
	**male (** ***n*** ** = 16)**	**female (** ***n*** ** = 36)**	
moderate and high progression (> 0.5 D/yr)	63%	58%	
no and low progression (≤ 0.5 D/yr)	38%	42%	
	*p* < 0.05	*p* < 0.01	
**older than 10 years at baseline**			
	**male (** ***n*** ** = 46)**	**female (** ***n*** ** = 64)**	
moderate and high progression (> 0.5 D/yr)	22%	23%	
no and low progression (≤ 0.5 D/yr)	78%	77%	
	*p* < 0.05	*p* < 0.05	

No and low progression was accounted for successful treatment, moderate and high progression was accounted for insufficient treatment effect. The *p*-values correspond to the statistical binomial tests of the percentage of treatment success in the eight cohorts against the percentage of treatment success in the total population

Findings of myopia progression correspond to those of AL growth rates in the nomogram (Figs. 3 and 4; Table 2), both for the classification based on baseline axial length and age: male eyes with a moderate baseline AL had a significantly higher proportion of non and low progressors than total population (89% > 65%, *p* < 0.01), while male eyes with high baseline AL had a lower proportion of non and low progressors (51% < 65%, n.s.). Female eyes below and above the 98th percentile showed similar proportion of non and low progressors (68% and 62%); both not significantly smaller or higher to that of the total population (65%). Compared to total population, eyes of younger children had significantly lower proportions of non and low progressors in both sexes (male: 38% < 65%, *p* < 0.05; female: 42% < 65%, *p* < 0.01), while eyes of older children had significantly higher proportions of non and low progressors in both sexes (male: 78% > 65%, *p* < 0.05; female: 77% > 65%, *p* < 0.05). Supplementary Fig. 1 offers another visualization of the data.

## Discussion

The 50th percentiles by Truckenbrod et al. [27] which we employed as criterion in this study correspond well to the modeled growth curve for emmetropic eyes by Jordan et al., who defined persistent emmetropes as showing refractive errors of more than − 0.25 D and less than + 1.00 D in both meridians at all study visits, each as measured by cycloplegic autorefraction [36]. Moreover, all patients we selected for treatment well fit into the category of children considered to have axial myopia as shown by their AL/CR ratios at baseline (Fig. 2). Accordingly, we consider our treatment criterion valid for the goals of this study.

### Treatment success with DIMS spectacle lenses

Within the first 12 months of treatment with DIMS spectacle lenses, 68% of male eyes and 64% of female eyes showed no or only low myopia progression, while 47% of male eyes and 45% of female eyes exhibited an AL growth rate in the range of physiological AL growth rate.

The ethnicity of the children was not taken into account because the differences in ethnicity, that has been reported so far, are more likely to be due to different environmental factors than to ethnicity itself [37]. Eyes of Asian children living in the Western cultures tend to develop like those of Caucasian children there [38]. It has also been reported that there is no difference in axial length between emmetropic Caucasian and emmetropic Asian eyes [39].

Notably, 55% of the male eyes and 63% of the female eyes had high baseline axial lengths above the 98th percentile of the epidemiologic reference curve of a German cohort. In the analysis of subgroups based on baseline AL, we found consistent findings for axial length growth rates and myopia progression: male eyes with moderate baseline AL were more likely to respond successfully to treatment than male eyes with high baseline AL. Female eyes also showed this trend, but without clinical or statistical significance. While this finding suggests that long eyes with a history of presumably rapid axial length growth continue to grow at an excessive rate, and are therefore more difficult to treat successfully, other studies suggest that rapid progression in the past does not necessarily mean rapid progression in the future [40].

Medians of AL growth rates in Table 3 also suggest that female eyes responded less well to treatment than boys, regardless of baseline axial length. We can only speculate about the causes and consequences of these sex differences in treatment success. One possible explanation is behavioral: girls may engage in more near-work activities, while boys may spend more time playing outdoor sports, where the exposure to natural light can help prevent or delay the onset of myopia [41–43]. Therefore, female eyes may not respond as successfully to treatment as male eyes do, regardless of baseline axial lengths. The observed differences in treatment effect could also be due to sex-specific growth patterns: From the reference curves [27] and in our own data, boys generally have longer AL than girls, but myopic girls’ eyes (i.e. above the 50th percentile) have a higher AL growth rate than boys’ eyes of the same age. To our knowledge, studies on myopia treatments have not focused on treatment efficacy of the different sexes yet, so this topic is still an area for further investigation. Due to the lack of reasonable data to date, it remains unclear whether boys respond better to myopia treatments than girls [40].

When classified by age, there were no such differences between the sexes. We also found congruent results for axial length growth rates and myopia progression (Table 4): based on both parameters, the eyes of younger children of both sexes responded successfully to treatment in a significantly lower proportion. Eyes of older children were significantly more likely to respond successfully to treatment.

### Application in daily practice

The long-term goal of myopia treatments is to maintain adult axial length at a physiological level and thus prevent complications. To achieve this, it is important to bring the excessive axial length growth rate, that leads to progressive myopia, to a physiological growth rate as quickly as possible.

We conclude from our results that treatment with the DIMS spectacle lenses alone may not be sufficient to achieve the treatment goal (1) in eyes with already high baseline axial length, and (2) in eyes of younger children. For these eyes, a combination with atropine eye drops should be considered. The combination of low-dose atropine eye drops and optical methods seems to enhance the inhibitory effect, also in combination with DIMS spectacle lenses [44, 45]. As cutoffs for high baseline axial lengths and younger age, we suggest axial lengths above the sex-specific 98th percentile and a baseline age of younger than 10 years old for European eyes. For other ethnicities, especially Asian eyes, these cutoffs may be different: the 98th percentile of German eyes, which indicates high myopia, is roughly equivalent corresponds to the 75th percentile of Asian eyes [27].

The median AL growth rate of eyes with moderate axial length and of older children reached the range of physiological growth within the first 12 months of DIMS treatment; for these children, the single treatment is sufficient.

### Study limitations

Limiting factors in this study are the relatively small cohort and subgroup size on the basis of which the conclusion are made. Since the data originates from a practice with specialized on progressive myopia in childhood, there is a large variation in the baseline values (especially baseline axial length) with partly extreme values, which are not necessarily representative for usual real-life cohort. The aim of this real-life study was to reproduce the results of Carly Lam’s placebo-controlled study with DIMS glasses (17). On the other hand, it was important to find out how the results change when patients outside the inclusion criteria of a study are included.

This means children who were younger than in the study and had higher myopia. A larger sample size would help in enhancing the statistical power and applicability our findings. Prospective, randomized controlled trials would be necessary to confirm a general consideration.

Additionally, cycloplegia was not consistently used to measure the objective refraction due to the procedure in the practice, so that the statements regarding dioptric myopia progression may have to be viewed with caution, as accommodation might influence dioptric values in children. However, it should be emphasized that the conclusions regarding myopia dioptric progression and axial length growth agree quite well.

## Conclusions

In a real-life observation of myopic eyes treated with DIMS spectacle lenses in a German cohort, the treatment goal of physiological AL growth rate was achieved in 46% of cases within the first 12 months of treatment. The treatment goal of no or only low myopia progression was achieved in 65% of cases.

A lower proportion of successful treatment was found in the groups with eyes with high baseline axial lengths and in younger children. For children with these eyes, a combination treatment should be considered.

To investigate the response to myopia treatments in the future, we advocate two things: first, to study boys and girls separately to detect potential differences in treatment response and behavior, and second, to assess treatment efficacy in terms of axial length growth rates based on physiological axial length growth rather than comparison to an untreated control group, which increasingly confronts researchers with an ethical dilemma.

## Electronic supplementary material

Below is the link to the electronic supplementary material.

Supplementary Material 1

Supplementary Material 2

## Data Availability

No datasets were generated or analysed during the current study.

## Abbreviations

AL: axial length
AL/CR ratio: axial length/corneal radius of curvature ratio
DIMS: defocus incorporated multiple segments
D/yr: diopter per year
mm/yr: millimeter per year
RCT: randomized controlled trial
SD: standard deviation
yrs: years

## References

[1] Zadnik K, Sinnott LT, Cotter SA, et al. Prediction of juvenile-onset myopia. JAMA Ophthalmol. 2015;133(6):683–9. 25837970 10.1001/jamaophthalmol.2015.0471 PMC4607030

[2] Saw S-M, Gazzard G, Shih-Yen EC, Chua W-H. Myopia and associated pathological complications. Ophthalmic Physiological Optics: J Br Coll Ophthalmic Opticians (Optometrists). 2005;25(5):381–91. 10.1111/j.1475-1313.2005.00298.x 16101943

[3] Flitcroft DI. The complex interactions of retinal, optical and environmental factors in myopia aetiology. Prog Retin Eye Res. 2012;31(6):622–60. 22772022 10.1016/j.preteyeres.2012.06.004

[4] Wong TY, Ferreira A, Hughes R, Carter G, Mitchell P. Epidemiology and disease burden of pathologic myopia and myopic choroidal neovascularization: an evidence-based systematic review. American journal of ophthalmology. 2014;157(1):9–25.e12. Published October 5, 2013. 10.1016/j.ajo.2013.08.010 24099276

[5] Holden BA, Fricke TR, Wilson DA, et al. Global prevalence of myopia and high myopia and temporal trends from 2000 through 2050. Ophthalmology. 2016;123(5):1036–42. Published February 11, 2016. 26875007 10.1016/j.ophtha.2016.01.006

[6] Williams KM, Bertelsen G, Cumberland P, et al. Increasing prevalence of myopia in Europe and the impact of education. Ophthalmology. 2015;122(7):1489–97. Published May 13, 2015. 25983215 10.1016/j.ophtha.2015.03.018 PMC4504030

[7] Williams KM, Verhoeven VJM, Cumberland P, et al. Prevalence of refractive error in Europe: the European Eye Epidemiology (E(3)) Consortium. Eur J Epidemiol. 2015;30(4):305–15. Published March 18, 2015. 25784363 10.1007/s10654-015-0010-0 PMC4385146

[8] Ang M, Wong TY. Updates on Myopia: A Clinical Perspective, 1st ed. 2020. Singapore: Springer Singapore; Imprint: Springer, 2020, 1st ed. 2020.

[9] Carlà MM, Boselli F, Giannuzzi F et al. Overview on Defocus Incorporated Multiple Segments Lenses: A Novel Perspective in Myopia Progression Management. Vision (Basel, Switzerland). 2022;6(2). Published April 2, 2022. 10.3390/vision6020020 PMC9036268 35466272

[10] Lam CSY, Tang WC, Tse DY, et al. Defocus Incorporated multiple segments (DIMS) spectacle lenses slow myopia progression: a 2-year randomised clinical trial. Br J Ophthalmol. 2020;104(3):363–8. Published May 29, 2019. 31142465 10.1136/bjophthalmol-2018-313739 PMC7041503

[11] Jaskulski M, Singh NK, Bradley A, Kollbaum PS. Optical and imaging properties of a novel multi-segment spectacle lens designed to slow myopia progression. Ophthalmic Physiological Optics: J Br Coll Ophthalmic Opticians (Optometrists). 2020;40(5):549–56. Published August 18, 2020. 10.1111/opo.12725 32808381

[12] Gantes-Nuñez J, Jaskulski M, López-Gil N, Kollbaum PS. Optical characterisation of two novel myopia control spectacle lenses. Ophthalmic & physiological optics: the journal of the British College of Ophthalmic Opticians (Optometrists) 2023. Published February 4, 2023. 10.1111/opo.13098 36738176

[13] Lam CS, Tang WC, Lee PH et al. Myopia control effect of Defocus incorporated multiple segments (DIMS) spectacle lens in Chinese children: results of a 3-year follow-up study. Br J Ophthalmol 2021 Published March 17, 2021. 10.1136/bjophthalmol-2020-317664 PMC9340033 33731364

[14] Lam CSY, Tang WC, Zhang HY et al. Long-term myopia control effect and safety in children wearing DIMS spectacle lenses for 6 years. Scientific reports. 2023;13(1):5475. Published April 4, 2023. 10.1038/s41598-023-32700-7 PMC10073092 37015996

[15] Kaymak H, Graff B, Neller K, Langenbucher A, Seitz B, Schwahn H. Myopietherapie und Prophylaxe Mit „Defocus Incorporated multiple Segments-Brillengläsern. Der Ophthalmologe: Z Der Deutschen Ophthalmologischen Gesellschaft. 2021;118(12):1280–6. Published July 8, 2021. 10.1007/s00347-021-01452-y PMC8648703 34236491

[16] Lu Y, Lin Z, Wen L, et al. The Adaptation and Acceptance of Defocus Incorporated Multiple Segment Lens for Chinese Children. Am J Ophthalmol. 2020;211:207–16. Published December 13, 2019. https://pubmed.ncbi.nlm.nih.gov/31837317/31837317 10.1016/j.ajo.2019.12.002

[17] Lam CSY, Tang WC, Qi H et al. Effect of Defocus Incorporated Multiple Segments Spectacle Lens Wear on Visual Function in Myopic Chinese Children. Translational vision science & technology. 2020;9(9):11. Published August 5, 2020. 10.1167/tvst.9.9.11 PMC7442864 32879767

[18] Kaymak H, Neller K, Schütz S, et al. Vision tests on spectacle lenses and contact lenses for optical myopia correction: a pilot study. BMJ open Ophthalmol. 2022;7(1):e000971. Published April 5, 2022. 35464151 10.1136/bmjophth-2022-000971 PMC8984052

[19] Kaymak H, Mattern A-I, Graff B, et al. Sicherheit Von Brillengläsern Mit DIMS-Technologie Und Atropin in Der Kombinationstherapie Der Myopieprogression. Klin Monatsbl Augenheilkd. 2022;239(10):1197–205. Published August 25, 2022. 36008055 10.1055/a-1930-7116 PMC9578763

[20] Brennan NA, Toubouti YM, Cheng X, Bullimore MA. Efficacy in myopia control. Prog Retin Eye Res. 2021;83:100923. Published November 27, 2020. 33253901 10.1016/j.preteyeres.2020.100923

[21] Rozema JJ. Refractive development I: biometric changes during emmetropisation. Ophthalmic & physiological optics. J Br Coll Ophthalmic Opticians (Optometrists). 2023;43(3):347–67. Published February 5, 2023. 10.1111/opo.13094 36740946

[22] Chamberlain P, La Lazon de Jara P, Arumugam B, Bullimore MA. Axial length targets for myopia control. Ophthalmic Physiological Optics: J Br Coll Ophthalmic Opticians (Optometrists). 2021;41(3):523–31. Published May 5, 2021. 10.1111/opo.12812 PMC8252804 33951213

[23] Sankaridurg P, Berntsen DA, Bullimore MA, et al. IMI 2023 Digest. Invest Ophthalmol Vis Sci. 2023;64(6):7. 37126356 10.1167/iovs.64.6.7 PMC10155872

[24] Bullimore MA, Brennan NA, Flitcroft DI. The future of clinical trials of myopia control. Ophthalmic & physiological optics: the journal of the British College of Ophthalmic Opticians (Optometrists). 2023;43(3):525–533. Published March 10, 2023. 10.1111/opo.13120 36897281

[25] Tideman JWL, Polling JR, Vingerling JR, et al. Axial length growth and the risk of developing myopia in European children. Acta Ophthalmol. 2018;96(3):301–9. Published December 19, 2017. 10.1111/aos.13603 PMC6002955 29265742

[26] Klaver C, Polling JR. Myopia management in the Netherlands. Ophthalmic Physiological Optics: J Br Coll Ophthalmic Opticians (Optometrists). 2020;40(2):230–40. 10.1111/opo.12676 32202320

[27] Truckenbrod C, Meigen C, Brandt M et al. Longitudinal analysis of axial length growth in a German cohort of healthy children and adolescents. Ophthalmic & physiological optics: the journal of the British College of Ophthalmic Opticians (Optometrists). 2021. Published April 1, 2021. 10.1111/opo.12817 33792977

[28] Yam JC, Zhang XJ, Zhang Y et al. Three-Year Clinical Trial of Low-Concentration Atropine for Myopia Progression (LAMP) Study: Continued Versus Washout: Phase 3 Report. Ophthalmology. 2022;129(3):308–321. Published October 7, 2021. 10.1016/j.ophtha.2021.10.002 34627809

[29] Chamberlain P, Peixoto-de-Matos SC, Logan NS, Ngo C, Jones D, Young G. A 3-year Randomized Clinical Trial of MiSight lenses for Myopia Control. Optometry Vis Science: Official Publication Am Acad Optometry. 2019;96(8):556–67. 10.1097/OPX.0000000000001410 31343513

[30] Walline JJ, Walker MK, Mutti DO, et al. Effect of high add power, Medium Add Power, or single-vision contact lenses on myopia progression in children: the BLINK Randomized Clinical Trial. JAMA. 2020;324(6):571–80. 32780139 10.1001/jama.2020.10834 PMC7420158

[31] Pauné J, Fonts S, Rodríguez L, Queirós A. The role of back Optic Zone Diameter in Myopia Control with Orthokeratology lenses. J Clin Med 2021;10(2). Published January 18, 2021. 10.3390/jcm10020336 PMC7831104 33477514

[32] Kaymak H, Neller K, Graff B, et al. Optometrische Schulreihenuntersuchungen: Erste Epidemiologische Daten Von Kindern und Jugendlichen Der 5. Bis 7. Klasse. Ophthalmologe. 2022;119(Suppl 1):33–40. Published June 10, 2021. 34114061 10.1007/s00347-021-01427-z PMC8191721

[33] Mattern A-I, Neller K, Devenijn M et al. A comparison of optical biometers used in children for myopia control. Klinische Monatsblatter fur Augenheilkunde 2023. Published June 26, 2023. 10.1055/a-2117-9335 PMC10651350 37364606

[34] Hsu C-C, Huang N, Lin P-Y, et al. Risk factors for myopia progression in second-grade primary school children in Taipei: a population-based cohort study. Br J Ophthalmol. 2017;101(12):1611–7. Published March 18, 2017. 28315834 10.1136/bjophthalmol-2016-309299

[35] Chia A, Chua W-H, Cheung Y-B, et al. Atropine for the treatment of childhood myopia: safety and efficacy of 0.5%, 0.1%, and 0.01% doses (atropine for the treatment of myopia 2). Ophthalmology. 2012;119(2):347–54. Published October 2, 2011. 21963266 10.1016/j.ophtha.2011.07.031

[36] Jones LA, Mitchell GL, Mutti DO, Hayes JR, Moeschberger ML, Zadnik K. Comparison of ocular component growth curves among refractive error groups in children. Investig Ophthalmol Vis Sci. 2005;46(7):2317–27. 15980217 10.1167/iovs.04-0945

[37] Németh J, Tapasztó B, Aclimandos WA et al. Update and guidance on management of myopia. European Society of Ophthalmology in cooperation with International Myopia Institute. European journal of ophthalmology. 2021;31(3):853–883. Published March 5, 2021. 10.1177/1120672121998960 PMC8369912 33673740

[38] Rose KA, Morgan IG, Smith W, Burlutsky G, Mitchell P, Saw S-M. Myopia, lifestyle, and schooling in students of Chinese ethnicity in Singapore and Sydney. Archives of ophthalmology (Chicago, Ill.: 1960) 2008;126(4):527–530. 10.1001/archopht.126.4.527 18413523

[39] Ip JM, Huynh SC, Robaei D et al. Ethnic differences in refraction and ocular biometry in a population-based sample of 11-15-year-old Australian children. Eye (London, England). 2008;22(5):649–656. Published February 2, 2007. 10.1038/sj.eye.6702701 17277756

[40] Bullimore MA, Brennan NA. Juvenile-onset myopia-who to treat and how to evaluate success. Eye (London, England). 2023. Published September 14, 2023. 10.1038/s41433-023-02722-6 PMC10858167 37709925

[41] Pärssinen O, Kauppinen M. Associations of near work time, watching TV, outdoors time, and parents’ myopia with myopia among school children based on 38-year-old historical data. Acta Ophthalmol. 2022;100(2):e430–8. Published July 21, 2021. 34291573 10.1111/aos.14980

[42] French AN, Morgan IG, Mitchell P, Rose KA. Patterns of myopigenic activities with age, gender and ethnicity in Sydney schoolchildren. Ophthalmic Physiological Optics: J Br Coll Ophthalmic Opticians (Optometrists). 2013;33(3):318–28. Published March 4, 2013. 10.1111/opo.12045 23452023

[43] Xiong S, Sankaridurg P, Naduvilath T et al. Time spent in outdoor activities in relation to myopia prevention and control: a meta-analysis and systematic review. Acta ophthalmologica. 2017;95(6):551–566. Published March 2, 2017. 10.1111/aos.13403 PMC5599950 28251836

[44] Kinoshita N, Konno Y, Hamada N, Kanda Y, Shimmura-Tomita M, Kakehashi A. Additive effects of orthokeratology and atropine 0.01% ophthalmic solution in slowing axial elongation in children with myopia: first year results. Jpn J Ophthalmol. 2018;62(5):544–53. Published July 4, 2018. 29974278 10.1007/s10384-018-0608-3

[45] Huang Z, Chen X-F, He T, Tang Y, Du C-X. Synergistic effects of defocus-incorporated multiple segments and atropine in slowing the progression of myopia. Sci Rep. 2022;12(1):22311. Published December 24, 2022. 36566245 10.1038/s41598-022-25599-z PMC9789944

