# Supplementary material for: Effect of defocus incorporated multiple segments (DIMS) spectacle lenses on myopia progression in children: a retrospective analysis in a German real-life clinical setting
Source: BMC Ophthalmol. 2024 Sep 12;24:403. doi: 10.1186/s12886-024-03666-5 (PMC11391804; doi:10.1186/s12886-024-03666-5)
Supplement: Supplementary file 1 — Supplementary Material 1 [file 12886_2024_3666_MOESM1_ESM.docx]

**Supplementary Table 1** Baseline characteristics of the analyzed eyes, divided into the eight subgroups based on baseline axial length and age

| **moderate baseline axial lengths (below the 98^th^ percentile)** | | | |
| --- | --- | --- | --- |
| **male (n=30 eyes of 17 boys)** | age [years] | axial length [mm] | spherical equivalent [D] |
| range | 7.1 to 15.2 | 23.50 to 25.44 | -7.00 to -1.25 |
| mean ± SD | 11.9 ± 2.1 | 24.48 ± 0.52 | -3.22 ± 1.37 |
| median | 11.4 | 24.57 | -3.25 |
|  |  |  |  |
| **female (n=37 eyes of 20 girls)** | age [years] | axial length [mm] | spherical equivalent [D] |
| range | 7.4 to 16.9 | 23.28 to 24.89 | -5.50 to -0.625 |
| mean ± SD | 11.9 ± 2.7 | 24.08 ± 0.41 | -3.10 ± 1.11 |
| median | 11.3 | 24.07 | -3.00 |
|  |  |  |  |
| **high baseline axial lengths (above the 98^th^ percentile)** | | | |
| **male (n=36 eyes of 20 boys)** | age [years] | axial length [mm] | spherical equivalent [D] |
| range | 6.4 to 14.8 | 24.26 to 28.01 | -6.50 to -1.38 |
| mean ± SD | 11.0 ± 2.9 | 25.93 ± 0.81 | -4.54 ± 1.32 |
| median | 11.5 | 25.79 | -4.63 |
|  |  |  |  |
| **female (n=63 eyes of 35 girls)** | age [years] | axial length [mm] | spherical equivalent [D] |
| range | 7.2 to 15.3 | 23.61 to 26.32 | -8.75 to -2.00 |
| mean ± SD | 10.3 ± 2.1 | 24.96 ± 0.66 | -4.34 ± 1.63 |
| median | 10.5 | 24.97 | -4.25 |
|  |  |  |  |
| **younger than 10 years at baseline** | | | |
| **male (n=18 eyes of 9 boys)** | age [years] | axial length [mm] | spherical equivalent [D] |
| range | 6.4 to 9.7 | 23.66 to 26.32 | -6.375 to -1.50 |
| mean ± SD | 7.9 ± 1.1 | 25.10 ± 0.85 | -4.26 ± 1.27 |
| median | 7.7 | 25.05 | -4.25 |
|  |  |  |  |
| **female (n=36 eyes of 18 girls)** | age [years] | axial length [mm] | spherical equivalent [D] |
| range | 7.2 to 9.9 | 23.39 to 25.46 | -7.125 to -1.75 |
| mean ± SD | 8.5 ± 0.8 | 24.26 ± 0.55 | -3.42 ± 1.46 |
| median | 8.4 | 24.34 | -2.94 |
|  |  |  |  |
| **older than 10 years at baseline** | | | |
| **male (n=48 eyes of 24 boys)** | age [years] | axial length [mm] | spherical equivalent [D] |
| range | 10.3 to 15.2 | 23.50 to 28.01 | -7.00 to -1.25 |
| mean ± SD | 12.7 ± 1.6 | 25.33 ± 1.05 | -3.82 ± 1.56 |
| median | 12.6 | 25.21 | -4.00 |
|  |  |  |  |
| **female (n=64 eyes of 32 girls)** | age [years] | axial length [mm] | spherical equivalent [D] |
| range | 10.0 to 16.9 | 23.28 to 26.32 | -8.75 to -0.625 |
| mean ± SD | 12.2 ± 2.0 | 24.84 ± 0.73 | -4.14 ± 1.59 |
| median | 11.5 | 24.79 | -4.19 |
