# Supplementary figures and images for: Effect of defocus incorporated multiple segments (DIMS) spectacle lenses on myopia progression in children: a retrospective analysis in a German real-life clinical setting
Source: BMC Ophthalmol. 2024 Sep 12;24:403. doi: 10.1186/s12886-024-03666-5 (PMC11391804; doi:10.1186/s12886-024-03666-5)

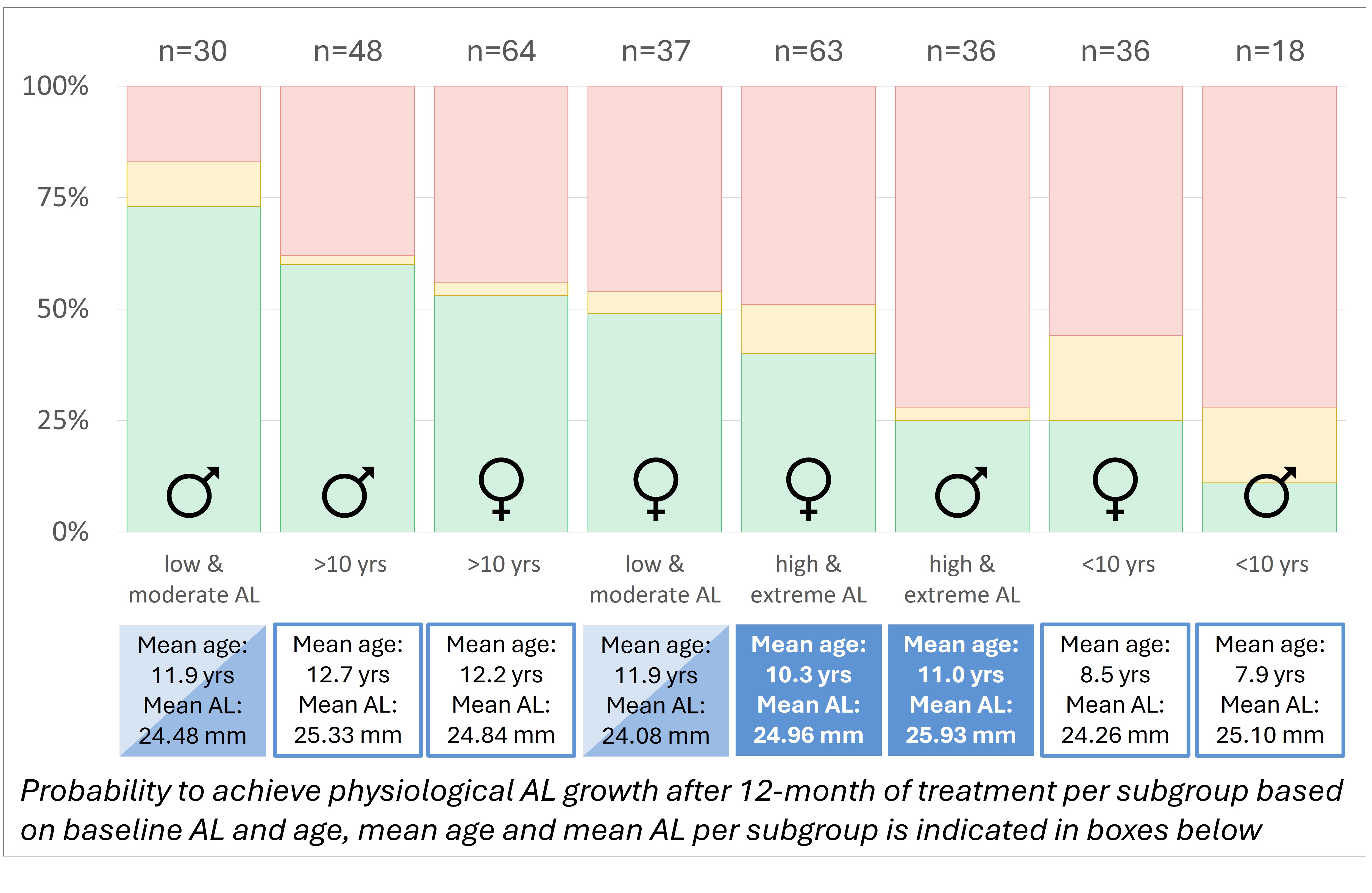

Supplement: Supplementary file 2 — Supplementary Material 2 [file 12886_2024_3666_MOESM2_ESM.jpg]
